# Users’ Support as a Social Resource in Educational Services: Construct Validity and Measurement Invariance of the User-Initiated Support Scale (UISS)

**DOI:** 10.3389/fpsyg.2016.01248

**Published:** 2016-08-23

**Authors:** Barbara Loera, Mara Martini, Sara Viotti, Daniela Converso

**Affiliations:** Department of Psychology, University of TurinTurin, Italy

**Keywords:** JD-R model, user support, social resources, educational services, confirmatory factor analysis, measurement invariance, UISS

## Abstract

Social support is an important resource for reducing the risks of stress and burnout at work. It seems to be particularly helpful for educational and social professionals. The constant and intense relationships with users that characterize this kind of service can be very demanding, increasing stress and leading to burnout. While significant attention has been paid to supervisors and colleagues in the literature, users have rarely been considered as possible sources of social support. The only exception is the Zimmermann et al.’s (2011) research, focused on customer support as a resource for workers’ well-being. This paper proposes the validation of the customer-initiated support scale developed by Zimmermann et al. (2011), translated into Italian and focused on educational services users (children’s parents), to measure the user support perceived by workers: the User-Initiated Support Scale (UISS). In Study 1 (105 teachers), which specifically involved educators and kindergarten teachers, the items and scale properties were preliminarily examined using descriptive analyses and exploratory factor analysis (EFA). In Study 2 (304 teachers), the construct and criterion validity and scale dimensionality were analyzed using confirmatory factor analysis (CFA). In Study 3 (304 teachers from Study 2 and 296 educators), measurement invariance (MI) was tested. The EFA results from Study 1 showed a one-factor solution (explained variance, 67.2%). The scale showed good internal coherence (alpha = 0.88). The CFA in Study 2 validated the one-factor solution (comparative fit index = 0.987; standardized root mean square residual = 0.054). Bivariate correlations confirmed construct validity; the UISS was positively associated (convergent) with user gratitude, and not associated (divergent) with disproportionate customer expectations. Regarding the criterion validity test, the UISS was strongly correlated with burnout and job satisfaction. The analysis of MI performed on the Study 3 data confirmed the equality of the parameters of the covariance structure model between the two samples of kindergarten teachers and educators. This research study offers a useful version of a tool for measuring a crucial, but often ignored, protective resource for all professionals working directly with people (patients, students, and service users) that can represent important sources of well-being, directly or indirectly lessening the negative impacts of job demands.

## Introduction

The renowned job demand-resources (JD-R) model (Bakker et al., 2003) describes the complex interactions that take place in the “dual process” induced by both requests and resources. Due to excessive demands, the first process results in the progressive exhaustion of workers, while the second process—the consequence of resource availability—may improve the ability to cope with demands, thus increasing motivation, satisfaction, and general participation at work. With the conservation of resources (COR) theory, Hobfoll (1989) explains how people strive to obtain, foster, and protect resources that can be delineated into four main categories—material, condition, personal, and energy resources. To better introduce the present research, it is useful to mention the social exchange theory (Foa and Foa, 1980), which underlines the resource exchange process that is activated during interpersonal relationships. From this perspective, every social exchange consists of taking and/or giving resources (money, goods, status, information, services, and love). Both resources theories have been widely considered in recent work and organization studies in the literature related to stress and burnout or to social support by co-workers and superiors, among other topics.

In all the models that are dedicated to the determinants of work-related stress, support from colleagues and/or supervisors is an important resource for protecting workers; conversely, when this support is absent or poor or when, on the contrary, colleagues and supervisors are bullying, workers’ psychological malaise can be worsened (Caplan, 1974; Johnson and Hall, 1988; Karasek and Theorell, 1990; Demerouti et al., 2001; Bakker et al., 2007; Arenas et al., 2015; Giorgi et al., 2016). In the literature, supervisors and colleagues are examined as possible sources both of support and of aggression. In service-sector organizations, conversely, users have rarely been considered as possible sources of support: the focus has been almost exclusively on the negative side of the relationship with “others.” Thus, disproportionate requests (Dormann and Zapf, 2004; Dudenhöffer and Dormann, 2013, 2015), customer mistreatment (Koopmann et al., 2015), or aggressive behaviors (LeBlanc and Kelloway, 2002; Viotti et al., 2015) have been considered to be factors that decrease job satisfaction and psychological well-being, contribute to developing stress, burnout, and the spiraling of negative exchanges between employees and customers (Groth and Grandey, 2012), and directly or indirectly enhance an employee’s intention to leave an organization (Lee and Ashforth, 1996; Jourdain and Chênevert, 2010). However, when service workers are asked why they have chosen their jobs (Maslach, 1982), they often declare their interest in “dealing with people” (Zimmermann et al., 2011, p. 31), thus implying that the motivational processes among service workers is often based on their interest in building a positive relationship with customers (users, patients, students, etc.). Contrary to the widely studied “negative side,” the relationship between service workers and users may then activate a positive gain spiral (Ferrara et al., 2013) and represent a resource, not just a request, for employees.

Consistent with the social exchange theory (Foa and Foa, 1980), in recent years some scholars have innovatively considered the hypothesis that service recipients can be important sources of support, and they have analyzed the direct and indirect effects of positive customer behavior on the positive affect of employees. For example, Converso et al. (2015a) and Martini et al. (2015) have shown that the perception of gratitude expressed by patients/customers was a relational resource useful to relieve the fatigue of daily commitment and to return significance to a professional’s work for healthcare workers and teachers, while Zimmermann et al. (2011) pointed out how sales workers’ behavior and customers’ behavior may activate reciprocal positive affective states. Thus, *customer-initiated support* is an “instrumental and emotional behavior that customers direct toward employees during the customer contact, making it easier to cope with service demands” (Zimmermann et al., 2011, p. 37). If different kinds of social support, such as material, behavioral, emotional, and informational support (Cohen and Wills, 1985), can be distinguished through feedback or attachment/integration, similar support can be offered by customers, too. According to the hypothesis posited by Zimmermann et al. (2011), customers may support employees in several ways: behaviorally by adapting their behaviors and expectations to the work process, emotionally by expressing appreciation for the employees’ work and becoming attached to them, and informationally by providing information and knowledge that can simplify the process.

In other words, customer support is a specific resource that can be considered for service organizations (Bakker et al., 2003). When it is derived from positive social exchanges (Foa and Foa, 1980), the COR theory (Hobfoll, 1989) indicates that it may counteract the loss of personal resources during the service interaction or promote a gain spiral through the positive climate of the relationship.

Zimmermann et al.’s (2011) customer-initiated support scale was developed in the retail sector. However, their suggestions may be even more important in the services sector where the social exchange between workers and the others occurs in an educational or healthcare relationship. On the one hand, employees are very motivated by “dealing with people” (Zimmermann et al., 2011, p. 31); on the other hand, the caring or educational process lasts longer than the customer/employee exchange. Preventing the loss of a personal resource from the users’ social support can be particularly useful because of the emotional and cognitively demanding nature of these types of occupations.

The present study was undertaken to investigate the impact of employee support in the early childhood education service sector. This sector has scarcely been considered in the stress or well-being literature (Hall-Kenyon et al., 2014) that has mainly studied teachers in primary school, secondary school, and college settings (Duncan, 1980; Byrne, 1991; Kelly and Berthelsen, 1995; Guidetti et al., 2015). Nonetheless, the early childhood education profession has specific features (Ohi, 2014) due to the very young age of the children whom the teachers care for (Converso et al., 2015b; Viotti et al., 2015). In the Italian context, based on the children’s age, further specificities exist between pre-school teachers (of 3- to 6-year-old children) and educators (of 0- to 3-year-old babies and toddlers). Educators have to care for infants and toddlers who are often not yet able to walk or talk, so these professionals are expected to engage in the physical work of lifting and the emotional work of cheering up children who suffer from their parents’ absence. Pre-school teachers have to support the children’s physical and cognitive development and autonomy, by playing and by developing school preparatory activities (e.g., painting, listening, or reading). Older children are able to speak clearly, walk, and provide for their own simple personal needs. Thus, pre-school teachers exert less physical effort than those who take care for babies and toddlers, and they also engage in more cognitive work with the children they teach, as part of applying the principles of child psychology.

Despite these differences, both the pre-school teachers’ and the educators’ educational work is perceived as being very demanding (cognitively, emotionally, and physically) because it requires playing a complex mixture of roles (Ohi, 2014; Nislin et al., 2016). As educators, they are responsible for sustaining the children’s overall development. As communicators, they should have the ability to interact effectively both with very young children and with parents. As care providers, they have to lift and carry children or bend, risking musculoskeletal disorders, as well as burnout (Koch et al., 2015). They also perceive themselves to be “pastoral care providers” (Ohi, 2014, p. 1013) as they have to support parents in their relationships with their children and in their personal crises. Thus, they may have to perform some non-teaching tasks (e.g., paperwork) that educators generally define as annoying and onerous. In addition to all these issues, pre-school teachers and educators perceive that they lack the resources they need and they are under hectic time pressures (Hall-Kenyon et al., 2014; Ohi, 2014).

To handle these requests, early childhood teachers can count on support from their colleagues and supervisors but, primarily, their relationship with the children is “the[ir] strongest source of satisfaction” (Jorde-Bloom, 1988, p. 118; Hall-Kenyon et al., 2014, p. 158). Thus, the relationship with users can enhance teachers’ well-being and reduce stress. Nevertheless, due to the specific nature of the users and the service, parents must be considered to be users as well. Ohi (2014) affirmed that, for teachers, developing a positive relationship with parents is “an enjoyable part of their work, even if it is also a daily challenge” (p. 1015). A positive relationship with parents can be defined as an alliance to support each child’s development; it is based on empathy, sharing educational objectives, frequent and honest exchange of information, reciprocal trust, and recognition of their roles, which are the dimensions of user support mentioned by Zimmermann et al. (2011).

The original scale developed by Zimmermann et al. (2011) measured the support from customers in market services. In this regard, our work aimed to measure the user support perceived in a helping-profession context by proposing a refined and validated version of the instrument developed by Zimmermann et al. (2011), which we renamed the User-Initiated Support Scale (UISS). We expected the UISS to adequately measure a single construct and to produce a valid measure, thus correlating with the only other available measure in our knowledge related to positive relational resources from users, the P-Grate scale, which considers the workers’ perceived gratitude (Martini et al., 2015). We anticipated that the measurement of user support would not be consistently correlated with the operators’ beliefs that users could be over-demanding and could make unrealistic requests beyond the professional role (Dormann and Zapf, 2004). Based on the hypothesis that user support would be a positive resource that would sustain well-being at work, we also expected that the presence of high support would be correlated with high work satisfaction and weak burnout symptoms.

Finally, since the meanings of the items might vary in terms of the function of the work’s content and its characteristics, even if the service offerings would remain the same, we decided to test the measurement invariance (MI) of the UISS between two different groups of professionals (kindergarten teachers and educators) operating in the same socio-educational service.

## Materials and Methods

### Procedure

The research was conducted at the Educational Service of the Municipality of Turin, Italy, between June 2013 and February 2014. The educational service includes both kindergarten and nursery schools for 0- to 6-year-old children that are residents of the city. The research design included a preliminary qualitative phase, consisting of 70 in-depth individual interviews with the more experienced kindergarten teachers and educators, as well as a quantitative phase using a questionnaire distributed to all kindergarten teachers and educators working in the Educational Service. The questionnaire was administered during work hours to all kindergarten teachers and educators who voluntarily agreed to participate in the research project. The subjects were asked to sign informed consent forms for the data analysis process. Anonymity of the data and the findings was ensured. The completed questionnaires were enclosed in blank envelopes and collected by researchers from the Department of Psychology, University of Turin.

### Ethics Statement

The present research study involved human beings in a data collection process in which participants were required to provide personal data concerning health information, personal opinions, and socio-demographical data. The research procedure was designed to conform to the provisions of the 1995 Declaration of Helsinki (as revised in Edinburgh in 2000), the Charter of Fundamental Rights of the European Union, the European Data Protection Directive (95/46/EC and following updates), and Italian laws on privacy and data protection (L. 196/2003). More specifically, data was collected and processed anonymously. The participants volunteered to participate in the research, and they were asked to sign an informed consent form in which they agreed to anonymously complete a questionnaire and allow the researchers to use the data for scientific purposes. No individuals unable to give informed content, vulnerable individuals or groups, or patients or minors were involved in the survey. The questionnaire used for data collection included a cover sheet that clearly explained the research aim, the voluntary nature of participation, the anonymity of the data, and the elaboration of the findings.

### Participants

In the first study (Study 1), 119 kindergarten teachers filled out the questionnaires; of these, 105 of the questionnaires were correctly completed and considered for the analysis. The 105 kindergarten teachers were all women, with an average age of 51.02 years (*SD* = 7.34).

Study 2 involved 320 kindergarten teachers; of these, 304 completely filled out the questionnaires, which were considered for the analysis. All of the 304 valid cases were women, with an average age of 51.92 years (*SD* = 7.25).

In Study 3, 308 educators in nursery schools filled out the questionnaire; 12 of the respondents returned incomplete questionnaires, which were discarded from the sample. The remaining valid questionnaires were from 296 educators, all women, with an average age of 47.59 years (*SD* = 7.59); most of the respondents were parents (77.5%) and 28.7% provided care for other people. On average, they worked 31.85 h per week (*SD* = 4.18). In this study, the 296 educators were compared with the 304 kindergarten teachers of the Study 2.

### Measures

Each participant filled out a self-report questionnaire. The introductory part consisted of socio-demographic and professional indicators. This was followed by a section that used scales to measure user-initiated support, the perceived gratitude of users, customer-related social stressors (CSS), and burnout. In addition, there was a single item on work satisfaction.

#### User-Initiated Support Scale

The UISS measures the support perceived by operators as a result of the users’ positive behavior. It applies the user–operator dyad to educational services. It is a modified version of the customer-initiated support scale that was initially developed for the employee–customer dyad (Zimmermann et al., 2011; original Cronbach’s α 0.82), which considered behavioral, informational, feedback, and emotional support. Following the International Guidelines on Test Adaptation (International Test Commission [ITC], 2005) the adaptation process has taken into full account the linguistic and the cultural differences among the populations for whom the adapted versions of the instrument are intended. The original scale (one item for each kind of support) was translated into Italian using the double-blind method, and an accurate translation was produced. The Italian version was back-translated by an individual whose native tongue is English; the original English scale and the back-translated scale were compared and the differences were discussed until a consensus was reached. The translated scale was then adapted for social operators, replacing “customer” with “user” in each item of the scale (**Table 1**). The five items on a 5-point Likert-response scale ranged from 1 = “I completely disagree” to 5 = “I completely agree.” The score of the scale was computed using the mean of the five items. A preliminary validation of the UISS involving health operators was proposed by Converso et al. (2015a). For the questionnaire intended for educational operators, the “users” referred to in the item formulations were the children’s parents. Because of the very young age of the direct users (children), parents (indirect users) should be considered as a possible source of support in the relationship with kindergarten teachers and educators. Before inserting the scale into the data collection tool, its adapted version was proposed to 27 kindergarten teachers and educators for a comprehension pretext; the items were considered enough clear by the subjects.

**Table 1:** Italian item translation.

User-Initiated Support Scale	Scala di Supporto offerto dagli utenti
1. The users adapted my working process	1. Gli utenti trovano adeguato il mio modo di lavorare
2. The users facilitated the service conversation through his/her previous knowledge	2. Gli utenti facilitano la comunicazione relativa al servizio di cura con le loro conoscenze precedenti
3. The users trusted in my competencies	3. Gli utenti si fidano delle mie competenze
4. The users explicitly valued my work effort	4. Gli utenti riconoscono esplicitamente l’impegno che metto nel lavoro
5. The users and I were on the same wave length	5. Gli utenti ed io siamo sulla stessa lunghezza d’onda

#### P-Grate Scale

The P-Grate scale (Martini et al., 2015) measures the perception of user gratitude and the support function that user gratitude offers social operators. It consists of the two subscales of gratitude expression (three items, e.g., “Several users express gratitude for the care we offer them”) and gratitude as a source of support (five items, e.g., “Some users’ gratitude compensates for the efforts you make at work”). On a 5-point Likert answering scale, the items ranged from 1 = “I completely disagree” to 5 = “I completely agree.” The scores of the two subscales were obtained by calculating the respective mean scores of the three items and the five items. The original Cronbach’s α for each was 0.88 and 0.82, respectively.

#### Disproportionate Customer Expectations

The broader scale of CSS (Dormann and Zapf, 2004; Taddei and Vanni, 2008; Guglielmetti et al., 2014) represents the opposite construct of customer support. It includes the following four sub-dimensions: disproportionate customer expectations, customer verbal aggression, disliked customers, and ambiguous customer expectations. Only the disproportionate customer expectations subscale (eight items) was included in this present research study (original Cronbach’s α 0.86). This subscale aimed to measure the users’ excessive requests to operators as possible sources of stress. An example of an item is: “Our customers do not recognize the fact that we are very busy.” The items on the Likert response scale ranged from 1 = “not at all true” to 4 = “completely true.” To compute the score of the scale, we calculated the mean of the eight items.

#### Maslach Burnout Inventory

The Maslach Burnout Inventory–Educational Survey (Maslach and Jackson, 1986; Sirigatti and Stefanile, 1993; Loera et al., 2014) measures operators’ perceptions of burnout using three subscales: Emotional Exhaustion (EE; eight items, e.g., “I feel used up at the end of the workday”), Depersonalization (DP; five items, e.g., “I feel I treat some recipients as if they were impersonal objects”), and Personal Accomplishment (PA; seven items, e.g., “I can easily understand how my recipients feel about things”). The original (Sirigatti and Stefanile, 1993) Cronbach’s α for each of these subscales was 0.87, 0.68, and 0.76, respectively. The responses were given on a Likert scale ranging from 0 = “never” to 6 = “every day.” The scores of the subscales were obtained by computing the mean scores of the eight, five, and seven items, respectively.

#### Job Satisfaction

To measure work satisfaction, we used a single item from the Organizational Health Questionnaire (OHQ; Avallone and Paplomatas, 2005): “How much do you feel satisfied about your work [referring to the last three months?].” The responses were given on a Likert scale ranging from 1 = “not at all” to 10 = “completely.” For its brevity, a single item is particularly suitable in a study that takes negative (EE and DP) and positive (PA) variables into account.

### Data Analysis

The items and scale properties were preliminarily explored using a descriptive analysis of the sample of 105 kindergarten teachers who participated in Study 1. Scale reliability was assessed with Cronbach’s coefficient, while the contribution to internal consistency at the item level was evaluated by the item–total correlations. Exploratory factor analysis (EFA) was used to check the factorability of the items correlation matrix.

The confirmatory factor analysis (CFA) model was specified in the data collected in Study 2 for testing item validity and scale dimensionality. CFA is a theory driven technique; it is applied to test hypotheses about the factor structure of the measurement instrument (Kline, 2011). It is recommended over EFA when there is an *a priori* hypothesis regarding dimensionality, as it allows testing of whether the empirical data fit an assumed structure (Floyd and Widaman, 1995). In this case, we assumed that UISS adequately measured a single latent dimension of support from service users.

Since data generally do not have univariate normal distributions let alone multivariate normal distribution, as requested in CFA models, it is dangerous to apply a normal theory-based estimation method on Pearson’s correlations. In trying to solve this problem, Jöreskog and Sörbom (1996a) found that, regardless of sample size and population correlation, polychoric correlations were the most consistent and robust estimator. Moreover, it has been demonstrated that the use of polychoric correlations provides a more accurate reproduction of the measurement model (Holgado–Tello et al., 2010). Following Jöreskog and Sörbom (1996b), after the normality of the items distributions was assessed, the model estimation was produced by applying the maximum likelihood method to the polychoric correlation matrix to correct the non-normal distribution of the UISS items in the sample by using the corresponding asymptotic covariance matrix.

Model fit was assessed by considering the goodness-of-fit index (GFI), the comparative fit index (CFI), and the standardized root mean square residual (SRMR). The GFI measures the amount of variance explained by the model, while the CFI indicates the relative amount of variation accounted for by the model by comparing its fit with a baseline model (null model). Both indices range from 0 to 1, and values higher than 0.90 are considered to be indicators of a good model fit (Bentler, 1995; Hoyle, 1995). The SRMR is a measure of the mean absolute correlation residual, that is, the overall difference between the observed and predicted correlations. An SRMR value less than 0.05 indicates a good fit (Byrne, 1998; Diamantopoulos and Siguaw, 2000); an SRMR value less than 0.08 is considered acceptable (Hu and Bentler, 1999).

The construct and criterion validity of the UISS were assessed using bivariate correlations. Specifically, the correlation with a similar construct (user gratitude) was analyzed to test the convergent validity of the UISS, and the correlation with a different construct (disproportionate customer expectations) was observed for divergent validity. The criterion validity of the UISS was evaluated by relating the user support score with well-being at work (work satisfaction and burnout). To improve immediacy and to compare the results, the mean of individuals’ answers was calculated for each scale, in order to maintain the same short range of the items response scale and the same range of scores regardless of the specific length of each instrument.

Finally, a multigroup confirmatory factor model was estimated to test the MI in the samples of kindergarten teachers and educators collected in Study 2 and Study 3. MI tests enable one to assess whether a scale is measuring the same latent variable in all of the significant population subgroups because comparisons and analyses of scores can be acceptable and meaningful only if the assumption of invariance is confirmed (Meredith, 1993; Reise et al., 1993; Widaman and Reise, 1997; Vandenberg and Lance, 2000). Toward that end, six hypotheses were tested using an equal number of structural equation models:

(1) The construct operationalization is valid in each group (configural invariance: equal number of latent factors and the same pattern of factors loadings);
(2) The respondents attribute the same meaning to the items, i.e., to the measured latent construct (metric invariance: factor loadings restricted to be invariant across groups);
(3) The respondents attribute the same meaning to the latent construct, as well the same meaning to the levels of the underlying items (scalar invariance: factor loadings and items intercepts restricted to be equal across groups);
(4) The latent construct is measured identically across groups, i.e., the explained variance of each item is the same in both groups (uniqueness invariance: factor loadings, items intercepts, and residual variances restricted to be equal across groups);
(5) The groups have the same mean for the latent construct (structural invariance in terms of factor means: factors means restricted to be equal across groups);
(6) The groups have the same range of scores for the latent factor (structural invariance in terms of factor variances: factor variances restricted to be equal across groups).

Testing for measurement (item loadings, intercepts, and uniqueness) and structural invariances (factors means and variance) was conducted using the scaled chi-squared difference test for nested models (Satorra and Bentler, 1994, 2001). The analyses were performed using PRELIS, LISREL 8.72, and IBM SPSS Statistics 22.0 software programs.

## Results

### Study 1: Descriptive Analysis, Reliability, and Exploratory Factor Analysis

The five items of the UISS did not have a normal distribution (see **Table 2**). Even if the values for items 2 and 5 were not very sharp, all the item distributions showed negative skewness and had positive kurtosis, particularly items 1 and 3. Both the Kolmogorov–Smirnov (KS) and the Shapiro–Wilk (SW) normality tests consistently suggested rejecting the hypothesis of normality of the distributions for all items.

**Table 2:** Descriptive analysis of the User-Initiated Support Scale items.

User-Initiated Support Scale	*M*	*SD*	Skewness	Kurtosis	KS	*p*	SW
1. The users adapted my working process	3.80	0.90	-0.87	1.21	0.30	0.000	0.83
2. The users facilitated the service conversation through his/her previous knowledge	3.20	0.96	-0.28	0.15	0.24	0.000	0.89
3. The users trusted in my competencies	3.96	0.83	-0.95	1.84	0.30	0.000	0.80
4. The users explicitly valued my work effort	3.83	0.99	-0.88	0.84	0.25	0.000	0.85
5. The users and I were on the same wave length	3.26	0.93	-0.17	0.06	0.22	0.000	0.90

Since the items of the scale did not have normal distribution, the EFA model was estimated using the generalized least squares extraction method. The mono-factorial solution explained the 67.18% variance. As shown in **Table 3**, Cronbach’s alpha on the UISS highlighted a satisfying internal coherence (0.88) that was quite close to the 0.82 Cronbach’s alpha noted by Zimmermann et al. (2011). Moreover, the item-scale correlation values were between 0.60 (item 2) and 0.75 (item 1), which was definitely above the cutoff value of 0.40 indicated by Nunnally (1967).

**Table 3:** EFA solution and reliability analysis of the User-Initiated Support Scale.

User-Initiated Support Scale	Factor loading	Corrected item-total correlations	Squared-multiple correlations	Cronbach’s alpha if item deleted
1. The users adapted my working process	0.850	0.750	0.644	0.837
3. The users trusted in my competencies	0.828	0.742	0.620	0.841
4. The users explicitly valued my work effort	0.797	0.724	0.583	0.844
5. The users and I were on the same wave length	0.763	0.715	0.562	0.846
2. The users facilitated the service conversation through his/her previous knowledge	0.641	0.602	0.395	0.873

Cronbach’s alpha	0.88			

### Study 2: Confirmatory Factor Analysis and Construct Validity

#### UISS Measurement Model

**FIGURE 1:**
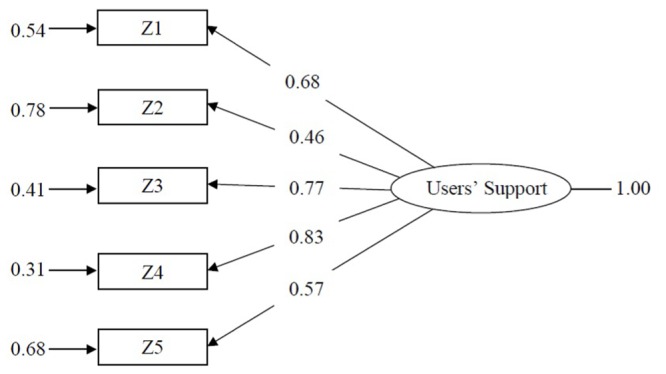
**CFA model for users support in educational service measured by UISS, 304 kindergarten teachers (standardized solution)**.

Because the UISS was built to measure one construct, the estimated CFA model was congeneric, with all items loaded on one factor. This model, illustrated in **Figure 1**, had a good fit and it performed satisfactorily. Moreover, if the minimum fit function chi-square was significant (χ^2^ = 30.26, *p* < 0.000), all the fit indices achieved the preselected cutoff values (GFI = 0.96; CFI = 0.98; SRMR = 0.05).

The item loadings were also all significant if item 2 seemed not quite efficient in measuring user support; its loading value was above 0.3, but its r-squared value was just 0.22, indicating the small amount of variance of this item when explained from the factor. In other words, for item 2, the amount of variance in common with the other items in the scale was 0.47. For comparison, the best scale item, in terms of loading and r-square, was item 4, which had at least 83% of variance in common with the other items.

#### Convergent and Discriminant Validity

The UISS had a strong positive correlation with the users’ expression of gratitude and with gratitude as a source of support. Both of these constructs were similar to user-initiated support since they represented different kinds of relational resources or different intentions of the meaning of user support. On the other hand, there was no correlation between disproportionate customer expectations and the expression of users’ negative behavior toward operators (**Table 4**). Therefore, the hypothesis about construct validity, both convergent and discriminant, was confirmed.

**Table 4:** Convergent and divergent validity: bivariate correlations with convergent and divergent constructs.

	1	2	3	4
1. User-initiated support	–			
2. Disproportionate customer expectations	-0.09	–		
3. Gratitude users’ expression	0.51^∗∗^	-0.04	–	
4. Gratitude as source of support	0.44^∗∗^	0.03	0.41^∗∗^	–
Mean	3.79	2.91	4.13	4.08
Standard deviation	0.62	0.93	0.81	0.76
Alpha	0.79	0.90	0.87	0.87

*^∗∗^*p* < 0.001.*

#### Criterion-Related Validity

Several authors (among the others: Etzion, 1984) have provided evidence showing that social support can reduce burnout (by reducing emotional exhaustion and depersonalization and increasing personal accomplishment) and enhance job satisfaction. Therefore, as a kind of social support at work, user support can, theoretically, be considered to be related to burnout and satisfaction at work. We used these constructs to test criterion-related validity. The UISS showed strong positive correlations with personal accomplishment and job satisfaction and a negative correlation with depersonalization (**Table 5**), confirming the hypothesis about criterion-related validity.

**Table 5:** Criterion-related validity: bivariate correlations with concurrent constructs.

	1	2	3	4	5
1. User-initiated support	–				
2. Emotional exhaustion	-0.03	–			
3. Depersonalization	-0.15^∗^	0.36^∗∗^	–		
4. Personal accomplishment	0.34^∗∗^	-0.08	-0.18^∗∗^	–	
5. Job satisfaction	0.31^∗∗^	0.53^∗∗^	-0.23^∗∗^	0.26^∗∗^	–
Mean	3.79	2.31	0.51	4.65	7.10
Standard deviation	0.62	1.48	0.73	0.98	1.85
Alpha	0.79	0.90	0.60	0.77	–

*^∗^p < 0.05; ^∗∗^p < 0.001.*

### Study 3: Measurement Invariance

**FIGURE 2:**
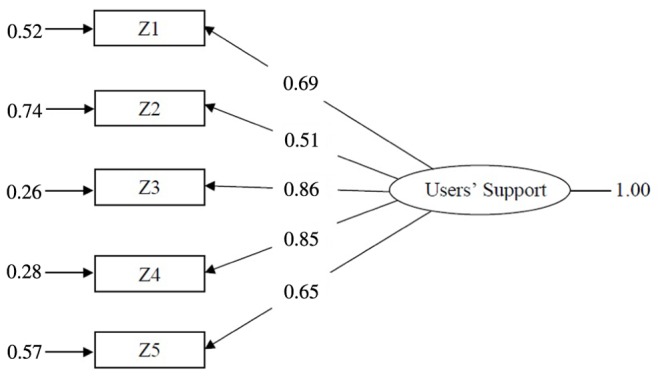
**CFA model for users support in educational service measured by UISS, 296 educators (standardized solution)**.

The socio-educational service examined in the present research study included two groups of workers that were in contact with the same users but that were involved in different tasks and relationships. These differences led to testing whether the UISS could have the same psychometric performance in the two workers’ subpopulations, that is, whether the measurement model estimated in the kindergarten teachers’ subsample might be valid and useful for the educators subsample, too. The fit for the model that was estimated by using the educators’ sample was very satisfactory (GFI = 0.96; CFI = 0.97; SRMR = 0.04; χ^2^ = 32.70, *p* < 0.000), apart from root mean square error of approximation (RMSEA; 0.10), and apparently the model fit seemed very close to the one previously estimated for kindergarten teachers (**Figure 2**).

The invariance of the UISS was tested by imposing the same measurement model on samples of kindergarten teachers (Study 2, *N* = 304) and educators (Study 3, *N* = 296). The data had passed the more severe test of population invariance, which, in turn, might imply the equality of the parameters of the covariance structure model (factor loadings, unique variances, and factor variance). The chi-square test value for equality of covariance was 23.245, with a probability value of 0.08. Thus, we accepted the null hypothesis of invariance of the two observed covariance matrices. This might mean that the two subsamples of kindergarten teachers and educators had the same covariance matrix, namely, that they belonged to the same population of socio-educational workers, at least when considering the perception of user support of this service.

However, since the population equality might be considered only to be a preliminary indication that some MI existed between the groups (Vandenberg and Self, 1993; Vandenberg and Lance, 2000), we decided to consider four nested models (M1, M2, M3, and M4), with increasingly restrictive specifications, in order to identify the eventual sources of non-equivalence in the measurement. Moreover, because population equality might be a clue for MI, but is uninformative with respect to structural invariance (Byrne et al., 1989), we added two models (M5 and M6) to test the equality of the factor means and variances across the two groups of kindergarten teachers and educators.

The results (**Table 6**) showed that consistent with the tenability of the population equality hypothesis, MI was perfectly achieved. The differences among the nested models, calculated using the Satorra–Bentler scaled difference chi-square (Δχ^2^), were all insignificant, thus confirming that the measurement model had the same loadings, intercepts, and unique item variances between the two professional subgroups. The same conclusion was supported by the CFI that was substantially stable in the first five models; all the CFI differences were positive or had a maximum value of 0.01, indicating better fits or a minimal decrease in the CFI (Cheung and Rensvold, 2002).

**Table 6:** Tests of UISS measurement and structural invariance across teachers and educators.

Model and invariance	CFI	df	χ^2^	*p*	SBχ^2^	*p*	Δχ^2^	df	*p*	SRMR	RMSEA	RMSEA CI
M1. Configural	0.98	10	62.96	0.000	35.09	0.000				g1 = 0.04 g2 = 0.04	0.081	0.05; 0.09
M2. Metric	0.98	14	66.81	0.000	40.15	0.000	M_2_–M_1_ 2.88	4	0.579	g1 = 0.05 g2 = 0.05	0.079	0.05; 0.09
M3. Scalar	0.97	18	72.32	0.000	47.64	0.000	M_3_–M_2_ 5.47	4	0.242	g1 = 0.06 g2 = 0.05	0.074	0.05; 0.10
M4. Uniquenesses	0.98	23	80.26	0.000	49.79	0.000	M_4_–M_3_ 4.07	5	0.539	g1 = 0.05 g2 = 0.05	0.062	0.04; 0.09
M5. Factor means	0.97	24	105.61	0.000	67.08	0.000	M_5_–M_4_ 35.37	1	0.000	g1 = 0.05 g2 = 0.05	0.077	0.06; 0.09
M6. Factor variances	0.98	24	81.77	0.000	51.27	0.000	M_6_–M_3_ 5.18	6	0.521	g1 = 0.07 g2 = 0.07	0.062	0.04; 0.09

In examining the structure of the latent variable, the sixth model (M6) enabled us to determine that the factor measured by the UISS had the same variance in the two professional groups. In comparison to its more general model (M3), M6 produced an irrelevant increment of chi-square (Δχ^2^_M6-M3_ = 5.18, *p* = 0.521). Conversely, M5 assessed significant differences between the mean scores of the latent factor measured in the two groups (Δχ^2^_M5-M4_ = 5.18, *p* < 0.001), but these results were expected since the hypothesis about the equality of the item intercepts was confirmed, implying that the items’ mean scores were different only because of differences in the latent mean scores, and they were not due to the items’ specific factors.

In the end, all the estimated models performed well in terms of the CFI and the local (within groups) SRMR. However, the models fitted badly in terms of the RMSEA; in general, the RMSEA values were acceptable (<0.8), but never appreciable (<0.5).

## Discussion

Scholars have rarely examined the positive side of the relationship between service workers and service users. Consistent with the COR theory (Hobfoll, 1989) and with the social exchange theory (Foa and Foa, 1980), this side of the relationship may represent an important social resource, may contribute to supporting service workers, and may activate positive gain spirals. Zimmermann et al. (2011) developed the customer-initiated support scale, which, to our knowledge, is the only scale specifically devoted to measuring users’ cognitive and emotional support, but they only referred to the retail sector. In the social/healthcare service sector previous studies have highlighted the importance of the perceived gratitude expressed, for example, by patients to enhance job satisfaction or to buffer burnout (Converso et al., 2015a). Gratitude may be considered to be a source of emotional support, while Zimmermann et al. (2011) also considered behavioral, informational, and feedback support. Thus, this present work aimed to validate UISS, a revised version of the scale developed by Zimmermann et al. (2011), to evaluate the impact of support for workers in the social (educational) services. Three studies involving kindergarten teachers were presented in this paper to verify the mono-factorial structure of the instrument via EFA and CFA. UISS showed a satisfactory percentage of explained variance and internal coherence, very close to that of Zimmermann et al.’s (2011) original scale.

Moreover, both the construct and criterion validity were confirmed for UISS. Indeed, convergent validity was verified because other positive dimensions of the relationship with users (user gratitude) strongly correlated with user-initiated support, even if they were clearly distinct. Divergent validity was verified by the absence of any significant correlation with the perception that users’ requests exceeded the operators’ roles, which could be defined as a relational demand in the educational profession. Correlations with both the supposed positive and negative consequences of social support were then analyzed. Although, to the best of our knowledge, few studies have examined the protective role that user support plays in the risk of burnout and its enhancing role in job satisfaction, many studies have confirmed this function of social support (from supervisors and colleagues). Our research results confirmed the criterion validity hypothesis.

Based on these results, we can affirm that this study (and the resulting paper) provides a scale to measure a crucial, but largely under-explored, concept for research in the educational context—social support from users. Educational professionals have defined that support as the core and most important source of strain as well as a source of motivation and satisfaction (Ohi, 2014).

Thus, it was important to analyze whether there were differences between two groups of professionals. The UISS showed strict MI (metric, scalar, and uniqueness), as well as latent factor invariance, between kindergarten teachers and educators. These findings imply that the instrument was found to be equally valid and reliable for these two professional groups (Lance and Vandenberg, 2009). Moreover, the groups could be compared at the level of their respective, latent mean scores (Meredith, 1993).

### Limitations and Future Directions

The present study has some limitations. *In primis*, the sample size is quite limited, and the participants were from the same geographical area and organizational context. Future studies should involve a larger sample of educators and kindergarten teachers. Another drawback of this research is its cross-sectional design; a longitudinal design could more effectively verify the importance of a supportive relationship between service workers and service users and identify the possible consequences of the user-initiated support. The study, moreover, is focused on educational services professionals. In order to test the invariance of the scale using different kinds of social operators, further research can hypothesize about the inclusion of other groups of social professionals (e.g., healthcare-sector workers). The aim is to consider this specific, and not yet deeply studied, source of support in other working populations that deal with people, in order to verify the similarities and differences. Future research should also simultaneously involve workers and service users. In doing so, it will contribute to the analysis of the reciprocity between employees’ health/well-being and users’ perceptions of the quality of services (Dormann and Kaiser, 2002; Ferrara et al., 2013; Converso et al., 2015a), as well as the affective crossover process as originally hypothesized by Zimmermann et al. (2011).

Despite these limitations, the UISS scale proposed in this paper was found to be a simple, yet valid and reliable, instrument to measure user support in educational and social services. Thus, it is a useful scale for evaluating the type of relational resources that promote employee well-being. Further advantages of the UISS scale are its concise form and it ability to explore several facets of user support. Therefore, it is particularly suitable both for complex research designs that take into account numerous dimensions and for applied research in organizations that need brief and simple instruments.

## Author Contributions

BL, MM, SV, and DC equally contributed at the: conception and design of the work; the analysis of data for the work; acquisition and interpretation of data for the work; drafting the work and critically revising it; final approval of the version to be published; agreement to be accountable for all aspects of the work in ensuring that questions related to the accuracy or integrity of any part of the work are appropriately investigated and resolved.

## Conflict of Interest Statement

The authors declare that the research was conducted in the absence of any commercial or financial relationships that could be construed as a potential conflict of interest.
